# Repeatability of Contrast-Enhanced Ultrasound to Determine Renal Cortical Perfusion

**DOI:** 10.3390/diagnostics12051293

**Published:** 2022-05-23

**Authors:** Shatha J. Almushayt, Alisa Pham, Bethan E. Phillips, John P. Williams, Maarten W. Taal, Nicholas M. Selby

**Affiliations:** 1 Centre for Kidney Research and Innovation (CKRI), University of Nottingham, Nottingham DE22 3DT, UK; pham.alisa@gmail.com (A.P.); m.taal@nottingham.ac.uk (M.W.T.); nicholas.selby@nottingham.ac.uk (N.M.S.); 2 Department of Renal Medicine, Derby Hospitals NHS Foundation Trust, Derby DE22 3DT, UK; 3 MRC/Versus Arthritis Centre for Musculoskeletal Ageing Research, University of Nottingham, Nottingham DE22 3DT, UK; beth.phillips@nottingham.ac.uk (B.E.P.); john.williams7@nottingham.ac.uk (J.P.W.); 4 Department of Surgery and Anaesthetics, Royal Derby Hospital, Derby DE22 3NE, UK

**Keywords:** perfusion, kidney, contrast-enhanced ultrasound, repeatability

## Abstract

Alterations in renal perfusion play a major role in the pathogenesis of renal diseases. Renal contrast-enhanced ultrasound (CEUS) is increasingly applied to quantify renal cortical perfusion and to assess its change over time, but comprehensive assessment of the technique’s repeatability is lacking. Ten adults attended two renal CEUS scans within 14 days. In each session, five destruction/reperfusion sequences were captured. One-phase association was performed to derive the following parameters: acoustic index (AI), mean transit time (mTT), perfusion index (PI), and wash-in rate (WiR). Intra-individual and inter-operator (image analysis) repeatability for the perfusion variables were assessed using intra-class correlation (ICC), with the agreement assessed using a Bland–Altman analysis. The 10 adults had a median (IQR) age of 39 years (30–46). Good intra-individual repeatability was found for mTT (ICC: 0.71) and PI (ICC: 0.65). Lower repeatability was found for AI (ICC: 0.50) and WiR (ICC: 0.56). The correlation between the two operators was excellent for all variables: the ICCs were 0.99 for PI, 0.98 for AI, 0.87 for mTT, and 0.83 for WiR. The Bland–Altman analysis showed that the mean biases (± SD) between the two operators were 0.03 ± 0.16 for mTT, 0.005 ± 0.09 for PI, 0.04 ± 0.19 for AI, and −0.02 ± 0.11 for WiR.

## 1. Introduction

A growing body of evidence recognises that alterations in renal perfusion play a major role in the pathogenesis of different renal diseases, including the syndromes of acute kidney injury (AKI) [1,2] and chronic kidney disease (CKD) [3,4], as well as diabetic kidney disease [5,6,7] and in kidney transplant rejection [8]. Among the current promising available techniques for renal perfusion assessment in humans is arterial spin labelling magnetic resonance imaging (ASL-MRI), which has been validated against alternative measures of perfusion, including contrast agent-based methods [9]. However, this is limited by the lack of accessibility, high cost, and challenges of scanning acutely unwell patients. Colour and spectral Doppler ultrasound techniques are widely used for non-invasive assessment of renal blood flow. These techniques provide insight about blood velocity only in major renal vessels due to their inability to detect slow intracortical microvascular blood flow [10]. This limitation was improved with the introduction of a new Doppler technique (Microvascular Doppler ultrasound), which has improved the ability to delineate renal microvasculature but does not provide quantitative measures of renal perfusion [11].

The interest in microvascular quantification stems from the fact that perfusion alterations can occur at the microcirculatory level without changes in large vessel blood flow [12], and since most of the blood entering the kidney supplies the renal cortex [13], direct assessment of microvascular perfusion can significantly expand our understanding of regional microvascular blood flow. Doppler-derived resistive index has been the focus of research for years as a potential marker of renal blood alterations but has been shown to be more reflective of systemic haemodynamics. Moreover, Doppler-derived indices were shown to correlate poorly with direct measurements obtained with an implanted flow probe in an experimental study [14]. Resistive index may still provide some prognostic information but not as a measure of microvascular perfusion [15,16,17,18,19].

Contrast-enhanced ultrasound (CEUS) is an alternative bedside technique for quantifying microvascular perfusion [20]. CEUS uses gas-filled microbubbles as an intravascular contrast agent (CA), which makes them suitable for microvascular perfusion assessment. Importantly, CEUS CA are not nephrotoxic and so are suitable for patients with renal insufficiency. With an increasing number of studies reporting the application of CEUS to assess renal perfusion [1,2,3,4,6,8,20,21,22], it is important to understand the performance of the technique, in particular the repeatability of the different quantitative perfusion measures that are generated. This is essential to supporting its translation to patient-based studies and before it can be introduced into clinical practice for this purpose.

Some studies have adopted standardised acquisition and analysis techniques to try to ameliorate variations in contrast perfusion variables [2,3,20,23]. One of these studies reported a high correlation coefficient between operators for two different perfusion variables (PI and AI) and moderate correlation for a separate variable (mTT) [23]. However, before more studies are conducted in patient cohorts, it is important to establish the repeatability of different CEUS-derived parameters in healthy cohorts, which has not been reported previously. Therefore, the aims of our study were (1) to determine the intra-individual repeatability of different measures of perfusion from renal CEUS taken under standardised conditions in healthy volunteers (HV) and (2) to test inter-operator repeatability of the process of image analysis.

## 2. Materials and Methods

This was a cross-sectional observational study designed to assess intra-individual and inter-operator variability of quantitative CEUS measures of renal perfusion. The study was approved by Research Ethics Committee (403-1910), and all participants provided written informed consent.

### 2.1. Participant Characteristics

Ten adults were recruited between February 2021 and April 2021. Inclusion criteria were healthy people above the age of 18 years, with no known kidney disease, diabetes, or hypertension and no known sensitivity to the CA (Sonovue^®^).

### 2.2. Study Procedures

Each participant attended the Royal Derby Hospital Centre for two renal CEUS scans within a 2-week period (to minimise significant physiological changes between the scans). No specific preparation was required from participants prior to the scan. Demographic and anthropometric details including age, gender, ethnicity, height, and weight were recorded, with blood pressure (BP) and heart rate (HR) also measured at each visit. All CEUS scans were performed by a single sonographer with CA administration performed by a medically qualified member of the research team. The primary outcome was intra-subject repeatability of CEUS-derived cortical perfusion parameters.

### 2.3. Renal CEUS Technique

CEUS scans were performed using a Philips iU22 ultrasound machine (Bothell, WA, USA), with contrast-specific software and a Philips C5-1 curvilinear transducer. Participants were instructed to lie on their left side, so the right flank was easily accessible for right kidney scanning. A 20 G cannula was placed in the participant’s right antecubital vein. SonoVue^®^ (Bracco, Milan, Italy) CA was prepared as per manufacturer’s instructions to yield 4.8 mL. This then was further diluted with 15.2 mL of 0.9% sodium chloride solution to yield a total volume of 20 mL. The CA syringe was then inserted into a dedicated infusion pump (VueJect^®^, Bracco, Milan Italy), which rotates the syringe to keep the contrast dilution agitated, preventing constituted bubbles from separating. The infusion line (length: 91 cm, internal diameter: 0.5 mm) was then primed and connected to the participant’s cannula, and the infusion rate was set at 3.3 mL/min.

CA infusion and imaging recording were started simultaneously. A period of two minutes was allowed for the CA to reach steady state, during which CA arrival to the kidney was visually observed. After this 2 min period, participants were instructed to hold their breath for 10 s and the transducer was held still in place. Five cycles of destruction/replenishment loops were captured, allowing participants to regain their breath between breath-holds. The destruction/replenishment loops involved a brief high mechanical index (MI) ultrasound pulse (flash) that caused complete CA destruction in the imaging field of view (FOV), followed by a replenishment phase when contrast re-entered the kidney. Upon completion of each assessment, recorded clips were exported to a hard drive in DICOM (digital imaging and communication in medicine) format for off-line analysis.

### 2.4. Ultrasound Machine Settings

Ultrasound machine settings were optimised at the beginning of each scan. The frame rate was 11 Hz and depth 14 cm. The focus depth was set below the kidney for a more unified beam towards the kidney. Time-gain-compensation (TGC) was set as follows from top to bottom: 53, 71, 71, 73, 73, 73, 73, and 73. The view displayed was set to dual contrast/B-mode, which was essential as contrast imaging supresses signals from background tissue to enhance the signals from inflowing bubbles. B-mode images were used to guide scanning and transducer position. Contrast resolution (C40) and MI (0.04) were the same on both sides, with higher gain on the contrast (96%) compared to tissue (80%) side. For destruction/replenishment loops, the low MI was 0.04 and the high MI was 1.07.

### 2.5. Image Acquisition

The right kidney was scanned in all patients but one, where the view was not satisfactory so the left kidney was scanned instead. A coronal or longitudinal imaging plane of the kidney was initially obtained with the same imaging plane used for all subsequent scanning. This was confirmed by reviewing first visit images and referring to notes on transducer position made at time of the first scan. The selection of the best view was based on the image quality of the visualised renal cortex that was clear of artefact, e.g., acoustic shadow from ribs.

### 2.6. Image Analysis

Analysis was performed using VueBox^®^ (Version 7.2.0.58362) Gastrointestinal (GI)I Perfusion Package (Bracco, Geneva, Switzerland) with this software calibrated to “Philips IU22, C5-1, C40, Map 2 Chr. Map Off” (scanner model, transducer, transducer resolution, and chr map) from the acquisition settings.

**Figure 1:**
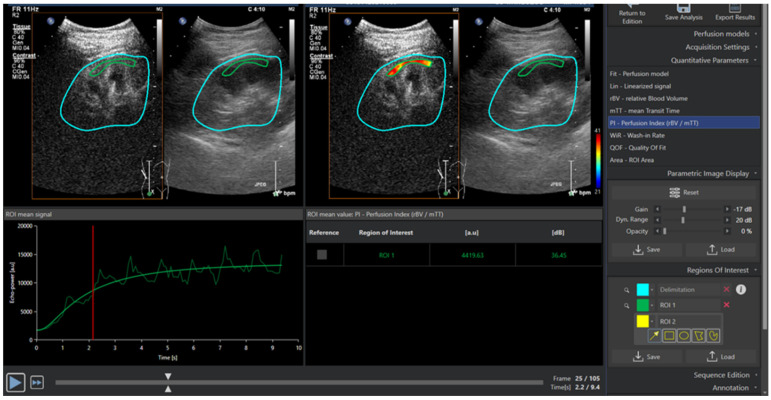
A quadrant view from the quantification software showing the drawn ROI around the renal cortex.

Each of the five recorded loops was analysed individually. First, using the clip editor, unwanted frames (beyond 10 s) were removed. A systematic approach was then adopted for region of interest (ROI) placement. A single ROI was drawn so that it included the largest possible area of renal cortex perpendicular to the ultrasound beam. In addition, the visualised renal cortex had to be free from any artefacts (acoustic shadow/enhancement), renal interlobar or arcuate vessels (verified by absence of bubbles right after the flash frame), inadequate insonification (flood of ultrasound waves), or excessive out-of-plane motion. VueBox^®^ measures the average intensity within the drawn ROI, so small ROIs were avoided to minimise local heterogeneities. VueBox then displays time–intensity curves (TIC) and a parametric image (heatmap), as shown in Figure 1. From the generated parametric image, the ROI was reassessed and adjusted as necessary to ensure there were detected signals within the drawn ROI for the desired perfusion parameters. Motion compensation settings were not used.

The ROI of the first loop was saved and loaded into subsequent loops for consistency. Minor adjustments to the ROI were performed as necessary, bearing in mind the same cortex depth and kidney pole. The generated data were analysed independently using GraphPad Prism^®^ version 9 (San Diego, CA, USA) so the method of generating the TIC could be specified. The non-linear one-phase exponential decay model (Y = (Y0-Plateau) × exp(−K × X)+ Plateau) was used, with Y0 constrained to Y0 = 0. The following perfusion parameters were derived for each loop individually: acoustic index (AI) (or plateau), which is the maximal intensity after reperfusion; mean transit time (mTT), which is the time needed after CA destruction to reach 50% of the maximal intensity; perfusion index, which is the ratio of AI to mTT; and wash-in rate (WiR) or K, which is the maximum slope. Each destruction-replenishment loop was analysed, and then, the median value was calculated for each parameter. A minimum of three loops with data of sufficient quality for analysis was required.

Image analysis was performed independently by two operators (S.J.A. and A.P.) to assess inter-observer variability.

### 2.7. Statistical Analysis

Analyses were performed using IBM SPSS^®^ (Version 27, New York, NY, USA). Normality of distribution was tested graphically. Non-normally distributed variables were log-transformed prior to analysis. Data are expressed as median and inter-quartile range (IQR) for non-normally distributed data. A *p*-value of <0.05 was considered statistically significant. The perfusion variables obtained from the two renal CEUS sessions for each participant were assessed for repeatability using an intra-class correlation (ICC) one-way model. Inter-observer variability was assessed using ICC two-way mixed model with absolute agreement. This was interpreted with reference to the criteria by Cicchetti [24], where an ICC of >0.75 is considered excellent, 0.60–0.74 is good, 0.4–0.59 is fair, and <0.40 is poor. Bland–Altman analyses were also performed and expressed as the mean difference, standard deviation and 95% limits of agreement.

The mean difference between the first and second scans was also calculated for each parameter. Associations between perfusion variables and subject characteristics were assessed using a two-tailed Pearson’s correlation for normally distributed data of continuous variables and with independent samples t-test for categorical variables. For non-normally distributed data, Spearman’s correlation and Mann–Whitney tests were used for continuous and categorical data, respectively. Sample size was determined based on the number of participants in most of the identified papers in the literature. Additionally, this sample size is adequate to demonstrate important points regarding the differences in repeatability between intensity-based and time-based measures that have relevance to the design of other studies using CEUS to assess renal perfusion.

## 3. Results

We recruited 10 participants (5 male and 5 female), with a median (IQR) age of 39 years (30–46) and body mass index (BMI) of 24.9 kg/m^2^ (22.3–25.9).

### 3.1. Perfusion Variables

**Figure 2:**
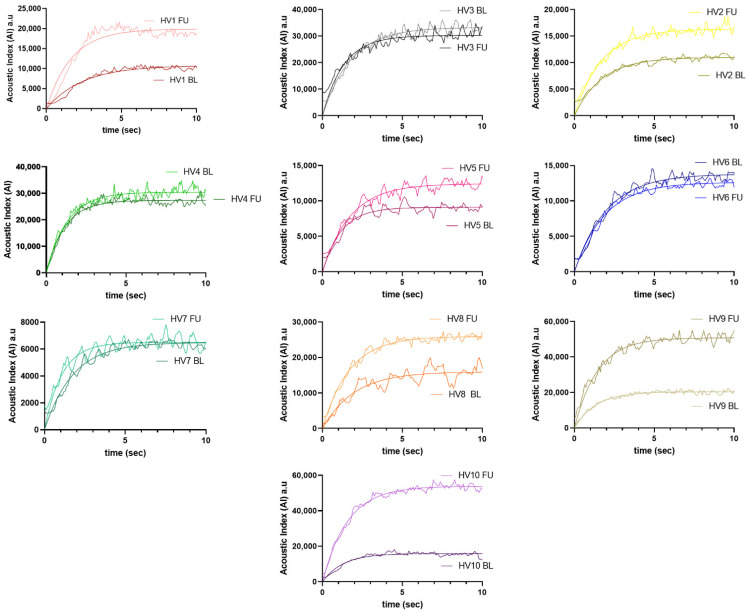
Time–intensity curves representing contrast replenishment in renal cortex.

Perfusion variables are shown in Table 1, and the mean and standard deviation (SD) of the difference between CEUS sessions in Table 2. Figure 2 shows the TIC for each participant in both visits. A summary description for participants’ blood pressure and heart rate in the two CEUS sessions is in Table 3.

### 3.2. Intra-Individual Repeatability

A good degree of repeatability was found for mTT (ICC: 0.71; 95% CI: −0.11 to 0.93; *p* = 0.03). The corresponding value for PI was (ICC: 0.65; 95% CI: −0.34 to 0.91; *p* = 0.06). Only fair, non-significant correlation values were found for AI (ICC: 0.50; 95% CI: from −0.89 to 0.94; *p* = 0.15) and WiR (ICC: 0.56; 95% CI: −0.67 to 0.89; *p* = 0.11). As seen in Figure 2, some individuals had TICs that were very similar between study sessions, which indicates good repeatability for both perfusion variables (AI and mTT), but others had clear differences, particularly in maximal intensity, reflecting less repeatability for AI. In these latter cases, the time to reach plateau appeared to vary less, which is consistent with the ICC analyses.

### 3.3. Inter-Operator Variability

**Figure 3:**
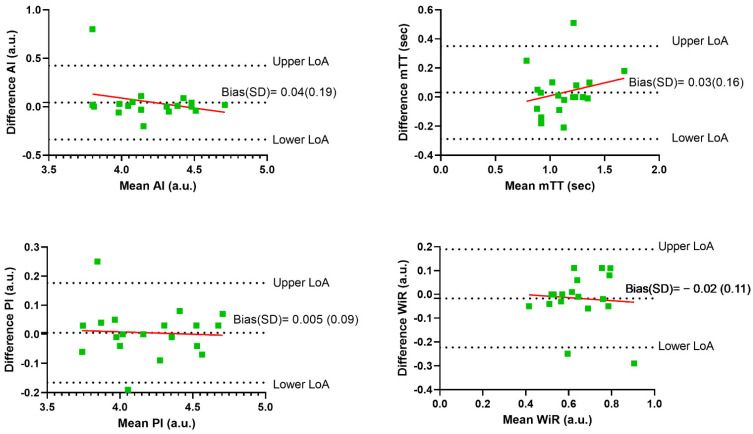
Inter-observer variability Bland–Altman plots; with linear regression line (red). LoA = 95% limit of agreement.

The correlation between the two operators were excellent for all values. For PI, ICC was 0.99; 95% CI: 0.96 to 1.00; *p* < 0.001. For AI, ICC was 0.98; 95% CI: 0.95 to 0.99; *p* < 0.001. For mTT, ICC was 0.87; 95% CI: 0.65 to 0.95; *p* < 0.001. For WiR, ICC was 0.83; 95% CI: 0.56 to 0.93; *p* < 0.001. Figure 3 shows the Bland–Altman plots for inter-observer agreement on each of the obtained perfusion variables. The mean difference between the two observers was close to zero for all variables with no evidence of systematic bias.

### 3.4. Relationship between Perfusion Variables and Personal Characteristics

There was no evidence of association between participant characteristics (age, BMI, HR, or BP) and perfusion variables. Similarly, there was no difference in perfusion values between genders (Table 4).

## 4. Discussion

In this study, we report intra-subject repeatability data from healthy volunteers for each of the measures of renal cortical perfusion generated from renal CEUS using the destruction/replenishment technique. The time-based measure of mTT had the best repeatability, whilst measures based on image intensity (AI) performed less well. The individual curves for each participant show results consistent with this (Figure 2), with some participants having very similar TICs between the two scans but others displaying large variations in AI (plateau) with less effects on mTT (half time). Additionally, we report a high degree of inter-operator agreement for all measures for the analysis process, suggesting that variation observed within individuals arises from differences in image acquisition. These results suggest that mTT is likely the most reliable measure for future work in which CEUS is used to assess renal perfusion in patient cohorts.

In the present study, we employed a systematic approach to tackle potential factors that could contribute to variations in the acquisition of CEUS perfusion measures. Image acquisition was performed with a standardised approach using consistent machine settings. Parameters obtained from a certain ROI depend on the attenuation properties of the tissues overlying it, through which ultrasound waves propagate. These vary not only with the type of the tissue but also across the population and may also be influenced by pathology [25]. Therefore, differences between study sessions that were seen in some individuals could have arisen from differences in the transducer position or scanning plane between study sessions, in addition to effects of breath-holding. Variation could also be attributed to the differences in the participants’ physiological status between the two study days. In this study, we assessed the relationship between BP, HR, and BMI with CEUS perfusion variables, and there was no significant correlation. However, other physiological factors that could potentially affect CEUS variables include hydration status [26], dietary salt intake [27], and tobacco use [28], which previously showed an effect on renal tissue oxygenation.

The analysis was performed with the ROI placed at the same depth and with those of the same size as far as possible. However, this was still subject to a degree of variability between the repeated flash/reperfusion loops. However, we have demonstrated that the analysis technique is repeatable with high inter-operator ICC values, and hence, the analysis process is unlikely to be a major source of variation.

Previous studies using renal CEUS for cortical perfusion quantification have used a variety of ROI sizes/numbers. Three small (5 × 5 mm^2^) ROIs have been used in some studies [2,20,29], whereas one larger ROI was used in others [1,3,23,30]. In our hands, we have found that adopting the largest ROI around visualised renal cortex helps avoid regional heterogeneity in renal perfusion and smooths out the generated TICs. The choice of a larger ROI could potentially average the effect of lateral shift variation, as observed by Ignee et al. [31], as VueBox^®^ calculates the average of intensities in a ROI. The good inter-observer agreement for all CEUS perfusion parameters reflects the minimisation of heterogeneity and reinforced our analysis approach. It is worth pointing out that we did not identify a reference ROI for normalisation as with renal tumour perfusion quantification studies, which means that a comparison of intensity-based variables between individuals becomes challenging. A reference ROI is normally placed within adjacent representative renal tissue at the same depth, an approach that was not possible with this method [32,33].

In accordance with our findings, a study by Harrois et al. [1] shows differences between time-based and intensity-based CEUS measures of perfusion in clinical settings. They reported that mTT was significantly increased in patients with septic shock who went on to develop severe AKI as compared with those who did not, with mean mTT values of 5.6 s and 3.4 s in the AKI and non-AKI groups, respectively. Notably, both groups had higher mTT ranges than seen in our healthy individuals (0.8–1.5 s), perhaps representative of disease-induced renal cortex hypoperfusion. However, in contrast to that reported for mTT, Harrois et al. [1] reported no difference in the intensity-based measures of regional blood volume (rBV) (equivalent to AI) and PI between AKI and non-AKI groups [1]. Other studies in animals have reported a higher variability of ultrasound measures of image intensity compared with time-dependent variables. For example, in a recent animal study by Liu et al. [34], variation in the repeated renal cortex CEUS perfusion parameters was assessed over 83 weeks in healthy dogs using a bolus CA technique. This study showed large coefficients of variation (COV) for intensity-based variables (of ~45%) compared with time to peak, which had a COV of only 14.7%. Similarly, Leinonen et al. [35] performed renal CEUS on healthy cats using a bolus technique and showed a significant change in the peak intensity variables with different cortical ROI depths and sizes. Taken together, these data suggest that time-based parameters are less susceptible to technical variation than those based on intensity.

There are some limitations to this study. First, the sample size was relatively small, and as this study was performed on healthy volunteers, the results may not be generalisable to patient cohorts. Future studies should report intra-subject repeatability for patients with kidney disease (e.g., CKD). Other potential physiological factors such as hydration status, dietary salt intake, or tobacco use were not evaluated nor controlled and, although unlikely to have been significantly different within individuals between study sessions, have potential to influence results. Finally, inter-operator variability was only assessed for the analysis process, not for image acquisition, another aspect that should be considered as scope for future work.

## 5. Conclusions

The current study reports the repeatability of CEUS-derived perfusion variables for renal cortical perfusion. Based on data from healthy individuals we conclude that the time-based variable (mTT) had good repeatability and is likely the most reliable measure for future studies in which CEUS is used to assess renal perfusion in patient cohorts and to assess changes in perfusion over time. The large intra-individual variation in intensity-based measures (AI) seen in some participants suggest that this parameter may not be suitable for this purpose.

## Figures and Tables

**Table 1:** CEUS-derived perfusion variables for scan1 and scan2; *n* = 10.

	mTT (Second)		AI (a.u.)		PI (a.u.)		WiR (a.u.)	
	Median (IQR)	Range	Median (IQR)	Range	Median (IQR)	Range	Median (IQR)	Range
Scan 1	1.07 (0.90–1.34)	[0.77–1.50]	14,120.50 (9872.13–22,728.00)	[6473.00–31,352.00]	10,302.64 (8647.33–23,680.44)	[5699.38–32,616.76]	0.65 (0.51–0.77)	[0.46–0.87]
Scan 2	1.07 (0.89–1.21)	[0.80–1.34]	22,083.50 (12,211.13–36,251.62)	[6411.00–52,191.00]	18,959.96 (9713.19–37,897.76)	[7682.51–55,390.67]	0.65 (0.57–0.78)	[0.52–0.87]

mTT = mean transit time; AI = acoustic index; PI = perfusion index; WiR = wash-in rate.

**Table 2:** Perfusion variables mean of the difference between scan1 and scan2; *n* = 10.

	mTT (sec)	AI (a.u.)	PI (a.u.)	WiR (a.u)
**Difference means (SD)**	0.06 (0.21)	−8993.35 (13,350.64)	−9653.75 (11,940.57)	−0.0150 (0.15)

mTT = mean transit time; AI = acoustic index; PI = perfusion index; WiR = wash-in rate.

**Table 3:** Patients’ cardiovascular measures on CEUS sessions; *n* = 10.

	CEUS1			CEUS2		
	HR (bpm)	SBP (mmHg)	DBP (mmHg)	HR (bpm)	SBP (mmHg)	DBP (mmHg)
**Median (IQR)**	60	121	82	54	122	81

HR = heart rate; bpm = beats per minute; SBP = systolic blood pressure; DBP = diastolic blood pressure.

**Table 4:** Association of gender with perfusion variables.

	Mean (SD) Female	Mean (SD) Male	*p*-Value
mTT (sec)	1.004 (0.16)	1.232 (0.30)	0.17
AI (a.u.)	202,796 (10,139.62)	12,360 (4254.49)	0.25
PI (a.u.)	20,636.48 (10,284.58)	10,486.44 (6151.23)	0.056
WiR (a.u.)	0.71 (0.101)	0.59 (0.169)	0.21

mTT = mean transit time; AI = acoustic index; PI = perfusion index; WiR = wash-in rate.

## Data Availability

The data presented in this study are available within the article.
